# Eagerness and Optimistically Biased Metaperception: The More Eager to Learn Others’ Evaluations, the Higher the Estimation of Others’ Evaluations

**DOI:** 10.3389/fpsyg.2018.00715

**Published:** 2018-05-15

**Authors:** Jingyi Lu, Hebing Duan, Xiaofei Xie

**Affiliations:** ^1^School of Psychology and Cognitive Science, East China Normal University, Shanghai, China; ^2^School of Psychological and Cognitive Sciences and Beijing Key Laboratory of Behavior and Mental Health, Peking University, Beijing, China

**Keywords:** metaperception, eagerness, positive emotion, self-evaluation, feelings as information

## Abstract

People frequently judge how they are viewed by others during social interactions. These judgments are called metaperceptions. This study investigates the relationship between eagerness to determine the evaluation of others and metaperceptions. We propose that eagerness, which reflects approach motivation, induces positive emotions. We apply feelings-as-information theory and hypothesize that positive emotions cause optimistic self-evaluations and metaperceptions. Participants in three studies interact with judges during a singing contest (Study 1), a speech (Study 2), and an interview (Study 3). Results corroborate that eagerness to learn the evaluation of others is overall related to optimistically biased metaperceptions. This effect is mediated sequentially by positive emotions, optimistic self-evaluations, and increased metaperceptions.

## Introduction

People form metaperceptions to establish how they are viewed by others during social interactions (Kenny and DePaulo, 1993). For example, interviewees judge how they are evaluated by interviewers, and speakers want to determine the audience’s evaluations of their performances. However, some individuals are more eager than others to learn how other people rate them. In general, people’s intuitions about the link between effort and output display a “more–more” pattern (i.e., more efforts, better outputs). For instance, students study hard to get a good TOEFL score. Tennis players practice long to win several tournaments. We aim to investigate if this pattern applies to the association between eagerness to learn others’ evaluations and accurate metaperceptions. In other words, we raise the question that whether high eagerness promotes or hinders accurate metaperceptions. Theoretically, seeking an answer to our question contributes to better understanding of the antecedents of accurate social predictions. Practically, it provides a way for accurate mind reading.

Research on social predictions has shown that inferences of others’ beliefs, thoughts, reactions, or characteristics are usually biased (e.g., Van Boven et al., 2005; Zhang and Epley, 2009; Klein and Epley, 2016, 2017). For instance, individuals overestimate the extent to which they are observed by others (Gilovich et al., 2000). People predict being judged more harshly by others than reality (Savitsky et al., 2001). Scholars agree to the idea that biased social predictions are derived from egocentrism that people fail to transcend from their own perspective when inferring others’ thoughts. For example, introspection illusion theory assumes that people judge themselves on the basis of their inner thoughts and feelings, whereas they judge others on the basis of others’ behaviors (Pronin, 2008). Spotlight theory states that people overestimate the brightness that the social spotlight shines on them than reality (Gilovich et al., 2000). As described in empathy gap theory, people are in a hot emotional state when judging themselves, whereas they are in a cold state when judging others. A lack of hot states hinders accurate social predictions. In sum, social predictions are egocentrically biased. On the basis of these theories, researchers find that inaccurate metaperceptions are caused by low perspective-taking abilities and low interpersonal sensitivity of a perceiver (Vorauer et al., 2009; Kenny et al., 2010).

Regarding our research question whether high eagerness promotes or hinders accurate metaperceptions, theories of egocentrism do not work because no direct evidence shows a link between eagerness and egocentrism. It urges us to adopt a new perspective to deconstruct how and why eagerness influences metaperceptions. We rely on a “motivation–emotion–cognition” perspective to find an answer.

Eagerness is a motivational factor characterized by approach motivation (Tauer and Harackiewicz, 1999), whereas forming metaperceptions is a cognitive process. Hence, finding a variable to build a bridge between these two factors is a solution to our question. Existing theories describe a close relationship among motivation, emotion, and cognition. On the one hand, feelings-as-information theory proposes that judgments depend on feelings even though these feelings are irrelevant to current judgments (Schwarz, 2012). On the other hand, emotion has a motivational root (Cacioppo et al., 1999). Therefore, we investigate the effect of eagerness (motivation) on metaperception (cognition) via emotions experienced by the perceivers.

Specifically, drawing on the idea that people with approach motivation are likely to experience positive emotions (Brockner and Higgins, 2001), we predict that eagerness will induce positive emotions. According to feelings-as-information theory, it is hypothesized that positive emotions will cause optimistic self-evaluations and metaperceptions. Assuming that judge’s rating is not influenced by eagerness level of the perceiver, the difference between metaperception and judge’s rating (metaperception – judge’s rating) will increase with eagerness.

### Metaperception and Self-Evaluation

Metaperceptions refer to the perceptions of the way others rate people (Kenny and DePaulo, 1993). Research has demonstrated that metaperceptions mainly rely on self-evaluations (Malloy and Janowski, 1992; Kenny and DePaulo, 1993; Nickerson, 1999). Hence, people overestimate the consistency between how they are viewed by others and by themselves. High self-evaluations are more likely to cause positive metaperceptions than low self-evaluations. Considering the positive correlation between self-evaluations and metaperceptions, we investigate how eagerness to learn the evaluations of others influences metaperceptions through self-evaluations.

### Eagerness Induces Positive Emotions

Eagerness is an incentive defined as how much people look forward to achieving a target (Lee and Chiou, 2015). Study has covered various types of eagerness, including eagerness to decide accurately (Lee and Chiou, 2015), to please others (Drake, 2014), and to change an undesirable status (Miller and Tonigan, 1996). Eagerness is characterized by approach motivation (Tauer and Harackiewicz, 1999). Strong eagerness correlates more with approach motivation than withdrawal motivation (Crowe and Higgins, 1997; Brockner and Higgins, 2001).

Eagerness to learn the evaluations of others reflects to what extent a person is motivated to know how they are rated by others. People who are eager to know the evaluations of others toward them tend to have an approach motivation more than a withdrawal motivation. Eagerness to learn others’ evaluations is distinguished conceptually from other concepts. (1) *Need for cognition*—this concept refers to a tendency to enjoy effortful thinking (Cacioppo and Petty, 1982) that is domain-general. Individuals with high needs for cognition have fun engaging in various types of thinking, not limited to how they are judged by others. In comparison, eagerness to determine others’ evaluations is further specific. (2) *Need for closure*—this concept is defined as a desire for clear rules and order compared with confusion and ambiguity (Webster and Kruglanski, 1995). Knowing others’ evaluations does not necessarily provide structure and eliminate confusions because people may not know the reason of these evaluations. (3) *Perception of doing well*. The perceptions of doing well and poorly can be associated with eagerness. For instance, a singer who felt that he/she sang well may be eager to know how the audience rated him/her. However, a singer who felt that he/she performed poorly may also be eager to know the audience’s evaluation for the improvement of his/her performance in the next show. In sum, eagerness to learn the evaluation of others is not identical to need for cognition, need for closure, or perception of doing well. In the following parts, the term “eagerness” will be used (short for eagerness to learn others’ evaluations).

Emotion is a key factor in shaping judgments. When investigating emotions, researchers highlight underlying motivational processes (Cacioppo et al., 1999). For instance, Watson et al. (1999) proposed that approach motivation underlies positive emotions, whereas withdrawal motivation underlies negative emotions. Although a counterexample exists (i.e., approach motivation underlies anger, which is a negative emotion; Carver and Harmon-Jones, 2009), most researchers agree that the majority of positive emotions are connected to an approach motivation orientation.

Given that approach motivation underlies positive emotions, people with approach motivation are more likely to experience positive than negative emotions due to a matching effect (Brockner and Higgins, 2001). In the study of Eddington et al. (2012), the approach and withdrawal motivations of participants were assessed, and their daily emotional experiences were recorded. Results showed that the participants with a high level of approach motivation tend to experience positive emotions (e.g., happy, satisfied, proud, and enthusiastic) more than negative ones (e.g., anxious, guilty, sad, and feel like a failure).

Thus far, the relationships between eagerness and approach motivation and between approach motivation and positive emotion have been clarified. Taken together, we predict that people who are eager to know how they are viewed by others are likely to experience positive emotions.

### Positive Emotions Promote Optimistically Biased Metaperceptions

Feelings-as-information theory elucidates that people attend to feelings as the sources of information for judgments even though these feelings are incidental and irrelevant to judgments (Greifeneder et al., 2011; Schwarz, 2012). For instance, people who judge a stranger ask themselves, “How do I feel?” If their feelings are positive, then they perceive the stranger as a nice person regardless of the source of feeling. Conversely, if their feelings are negative, then they will think otherwise. In the study of Schwarz and Clore (1983), participants who recalled a happy or sad event were asked to rate their life satisfaction. Life satisfaction was higher in the happy than sad condition. In another study, participants in a positive mood tended to interpret an ambiguous event as an opportunity, whereas participants in a negative mood tended to interpret the same event as a threat (Mittal and Ross, 1998).

Self-evaluations should be affected by feelings in accordance with feelings-as-information theory. People who judge themselves will attend to their feelings. The more positive the emotions they experience, the more optimistic their self-evaluations will be. Thus, eagerness to learn the evaluations of others should be positively correlated with positive emotions, self-evaluations, and metaperceptions.

As we try to go beyond metaperception itself and investigate biased metaperception, the judge’s rating will be used as a benchmark to test whether or not metaperception is accurate. An optimistic bias score was used to reflect the difference between metaperception and judge’s rating (metaperception – judge’s rating). Assuming that the judge’s ratings will not be influenced by the perceiver’s eagerness, we hypothesize that the optimistic bias score will increase with eagerness and positive emotions. A high score implies high overestimation if it is above zero, whereas it implies low underestimation if it is below zero. Whether it is overestimation or underestimation depends on many factors other than eagerness, including the judge’s rating (i.e., whether a judge adopts strict criteria). Thus, this case is not the focus of the present research. What we are interested in is how eagerness affects the magnitude of optimistically biased metaperceptions. In sum, our hypothesized pathway is as follows: eagerness → positive emotion → self-evaluation → metaperception → optimistically biased metaperception.

### Present Research

In this research, we attempted to investigate the relationship between eagerness to learn the evaluation of others and optimistically biased metaperception. The emotional routes that underlay such a relationship were also examined. Both field and laboratory studies were conducted.

We collected data from a singing contest in Study 1 (a field study). Singers rated their eagerness to know the evaluations of raters and made inferences about the evaluations of judges. Participants in Study 2 (a laboratory study) delivered a speech on a given topic to a judge. They indicated their eagerness to know the evaluations of the judge, emotional experiences, and self-evaluations and predicted the evaluations of the judge. In these two studies, both eagerness and metaperceptions were measured. Given the correlational nature, we attempted to manipulate eagerness to establish a causal relationship in Study 3 (a laboratory study). The participants were randomly assigned to a high or low eagerness condition. They were interviewed by an interviewer, whose evaluations they then judged.

This research project was approved by the Ethics Committee of School of Psychological and Cognitive Sciences, Peking University. Written informed consent was obtained from all participants. All materials and raw data will be made available by the authors, without undue reservation, to any qualified researcher.

## Study 1: Singing Contest

The relationship between eagerness and metaperception was tested in a natural setting. The Top 10 Campus Singers Contest in a university provided an opportunity to collect data. We invited singers to indicate their eagerness to learn the evaluations of the judges.

### Method

#### Participants

The first round of the Top 10 Campus Singers Contest for 2013 in a university was held on October 11, 12, 13, 18, 19, 25, 26, and 27, 2013. We were allowed to collect data on the last 3 days. A total of 111 singers (67 men, 44 women; *M*_age_ = 20.33 years, *SD* = 2.47) who took part in the last 3 days of the contest participated in this study. We assumed a small-to-medium effect size (*r* = 0.25) of the relationship between eagerness and metaperception (or optimistically biased metaperception), the power of this study is about 74%, given the sample size of 111 and α = 0.05 (Cohen, 1970).

#### Procedure and Materials

Each singer performed for 2 min. Five judges, who were either professors from the Academy of Opera or one of the Top 10 Singers from previous years, rated the singers’ performance from 0 (*very poor*) to 100 (*very good*). The average score was announced. The scores of the first five singers were announced after the performance of the sixth singer. The scores of the next five singers were announced after the performance of the 11^th^ singer. Three experimenters recorded these scores.

The experimenters invited the singers who completed their performance to complete a questionnaire before their scores were announced. The cover sheet of the questionnaire specified that this research was not related to the contest. The singers provided information regarding their contestant number, the number of instances they participated in the Top 10 Campus Singers Contest, gender (0 = men, 1 = women), and age. The participants then rated their eagerness (“I am eager to know the judges’ evaluations of me,” “I care about the judges’ evaluations of me,” and “The judges’ evaluations of me are important to me,” α = 0.81) and motivation to obtain a high score (“I strongly hope to advance to the next round,” “I strongly hope to obtain a high score,” and “I strongly hope to win the recognition of the judges,” α = 0.89) on a seven-point scale that ranged from 1 (*totally disagree*) to 7 (*totally agree*). The average scores were used. Two filler items were included to conceal the aim of the research (“I know well about my singing skill” and “I am confident of my singing”; 1 = *totally disagree*, 7 = *totally agree*). The participants then made inferences about the average score of the judges’ evaluations (i.e., metaperception; 0 = *very poor*, 100 = *very good*). Finally, the participants were debriefed and thanked. The average score of the judges and the participants’ ratings were paired according to their contestant number.

### Results and Discussion

We subtracted the average score of the five judges from the participants’ metaperception for each singer to reflect an optimistically biased metaperception. If the value is above zero, a high score indicates high overestimation. If the value is below zero, a high score indicates low underestimation. If the value equals zero, it indicates accurate metaperception. **Table 1** shows the descriptive statistics and correlation matrix among the variables. Metaperception was positively correlated with average score (*r* = 0.56, *p* < 0.001), indicating that participants, on average, were accurate in predicting others’ evaluations of themselves. In addition, eagerness was correlated with metaperception (*r* = 0.23, *p* = 0.015). The results indicated that metaperception increased with eagerness. However, the correlation between eagerness and optimistically biased metaperception did not reach conventional significance (*r* = 0.16, *p* = 0.102).

**Table 1:** Descriptive statistics and correlation matrix among variables (Study 1).

Variables	*M* (*SD*)	1	2	3	4	5	6	7
(1) Gender	–							
(2) Age	20.33 (2.47)	–0.02						
(3) Number of instances	1.28 (0.59)	0.11	0.19*					
(4) Eagerness	5.12 (1.43)	0.11	–0.09	0.09				
(5) Motivation to obtain a high score	5.26 (1.42)	0.14	–0.02	0.07	0.70***			
(6) Metaperception	84.59 (4.23)	0.02	0.02	0.23*	0.23*	0.27**		
(7) Average score	83.85 (3.67)	0.24*	0.15	0.39***	0.11	0.24*	0.56***	
(8) Optimistically biased metaperception	0.74 (3.74)	–0.21*	–0.14	–0.13	0.16	0.07	0.58***	–0.35***

*^∗^*p* < 0.05, ^∗∗^*p* < 0.01, ^∗∗∗^*p* < 0.001. Optimistically biased metaperception = metaperception – average score.*

Because optimistically biased metaperception was correlated with gender (*r* = -0.21, *p* = 0.024), a hierarchical regression was conducted to examine the relationship between eagerness and optimistically biased metaperception after controlling for the effect of gender. Gender was entered in the first step and eagerness was entered in the second step. As a result, optimistically biased metaperception increased with eagerness (β = 0.18, *p* = 0.049) after controlling for the effect of gender. This results revealed that eagerness was linked to optimistically biased metaperceptions.

It could be possible that eagerness correlated with optimistically biased metaperception because participants who believed that their performances were objectively better were more eager to learn their results. However, the fact that eagerness was not significantly correlated with the judges’ ratings (*r* = 0.11, *p* = 0.251) makes this alternative explanation implausible.

Limitations remained in this study. First, we did not control for the facial expressions, gestures, and tone of the judges because of the nature of the field study. These factors may covariate with the level of eagerness, which in turn influences metaperception. A strictly controlled laboratory study was conducted to solve this issue, where we ensured that the judge’s behavior was almost constant across participants. Second, emotion was not assessed in this study, making it hard to interpret why eagerness affected optimistically biased metaperception. Third, an alternative explanation existed that participants who had more positive self-evaluations tended to be more eager to know judges’ evaluations. To provide further evidence for our hypothesized pathway, we conducted a laboratory study on another type of interpersonal interaction, and excluded several confounding pathways.

Moreover, research has suggested that individuals with strong eagerness focus on a broad array of information (Tetlock and Boettger, 1989). In this sense, eagerness to know the evaluations of others promotes focus on both self and others. Meanwhile, focusing on others’ reactions (vs. one’s own behaviors) causes biased metaperceptions because information regarding ones’ own behaviors (vs. others’ reactions) is more objective. For example, in a study, some participants who were engaged in social interactions were provided with an opportunity to observe their own behaviors, whereas the rest were not allowed to do so. Participants who observed their own behaviors made more accurate metaperceptions than those who did not (Albright and Malloy, 1999). Therefore, in the next study, we also measured cognitive focus to examine its potential role in the relationship between eagerness and metaperception.

## Study 2: Speech

By conducting a laboratory study, we sought to provide further evidence on the relationships among eagerness, positive emotion, self-evaluation, and metaperception. Moreover, we also tested whether cognitive focus played a role in the relationship between eagerness and metaperception. Participants were recruited to deliver a speech on a given topic, and their performances were evaluated by a well-trained male judge. The participants indicated their eagerness, emotional experiences, cognitive focus, and metaperception.

### Method

#### Participants

A total of 102 university students were recruited, but 14 participants did not complete the speech as required^1^. The final sample comprised 88 participants (30 men, 58 women, *M*_age_ = 20.89 years, *SD* = 2.35). We assumed a medium effect size (*r* = 0.30) of the relationship between eagerness and metaperception (or optimistically biased metaperception), the power of this study is about 80%, given the sample size of 88 and α = 0.05 (Cohen, 1970).

#### Procedure and Materials

After the participants arrived at Laboratory A, an experimenter informed them that they would deliver a speech on an assigned topic for at least 3 min to a judge who would evaluate their performances. The topic was, “Are slack policies good or bad for university students?” The participants were given 5 min to prepare.

The participants were led to Laboratory B, where they delivered a speech to the same male judge who was not aware of the research aim. His clothing was constant across participants. One timer was placed in front of the judge and another in front of the participants. The judge did not talk with the participants during the speech. He nodded to half of the participants at the 30^th^ and 90^th^ second marks and shook his head at the 60^th^ and 120^th^ second marks. He shook his head at the other half of the participants at the 30^th^ and 90^th^ second marks and nodded at the 60^th^ and 120^th^ second marks. These head movements served as feedback from the judge and were standardized for all participants. The judge showed neutral facial expressions and avoided body language. The speeches were videotaped to record the length of speech delivered by each participant.

After delivering the speeches, the participants returned to Laboratory A where they completed the measures. They rated their eagerness (“I am eager to know the judge’s evaluation of me,” “I care about the judge’s evaluation of me,” and “The judge’s evaluation of me is important to me;” α = 0.78; 1 = *totally disagree*, 7 = *totally agree*) and indicated whether they felt nervous, anxious, happy, or excited during the speech (1 = *not at all*, 7 = *strongly*). The average score for nervousness and anxiety reflected intensity of negative emotion (α = 0.84), whereas that for happiness and excitement reflected intensity of positive emotion (α = 0.86). The participants also rated their cognitive focus during the speech (“During the speech, how much did you focus on your behaviors?” “During the speech, how much did you focus on the judge’s reactions?” 1 = *never*, 7 = *very frequently*). The former item reflected a focus on the self, whereas the latter reflected a focus on the judge.

Afterward, the participants inferred about the evaluation of the judge on nine dimensions, namely, public speaking skill, fluent speaking, accurate wording, modesty, confidence, enthusiasm, steadiness, easygoing trait, and optimism (0 = *very poor*, 10 = *very good*). Average rating was employed to reflect metaperception (α = 0.94). The participants also evaluated themselves on the aforementioned nine dimensions (α = 0.93). Thereafter, they indicated their gender (0 = men, 1 = women) and age. Finally, the researchers debriefed and paid the participants.

The judge stayed in Laboratory B and rated each participant based on the nine dimensions (α = 0.91). An average rating was adopted to reflect the evaluation of the judge. The experimenter paired the evaluations of the judge and the ratings of the participants.

### Results and Discussion

We subtracted the evaluation of the judge from the metaperception of participants to reflect an optimistically biased metaperception. **Table 2** displays the descriptive statistics and correlation matrix among variables. Metaperception was positively correlated with judge’s evaluation (*r* = 0.37, *p* < 0.001), indicating that participants, on average, made accurate predictions. Furthermore, consistent with existing findings, self-evaluation was highly correlated with metaperception (*r* = 0.82, *p* < 0.001), showing that participants formed metaperceptions on the basis of self-evaluations.

**Table 2:** Descriptive statistics and correlation matrix among variables (Study 2).

Variables	*M* (*SD*)	1	2	3	4	5	6	7	8	9	10	11
(1) Gender	–											
(2) Age	20.89 (2.35)	–0.16										
(3) Length	222.13 (62.86)	–0.01	0.15									
(4) Eagerness	4.31 (1.27)	–0.14	–0.05	0.14								
(5) Negative emotion	3.98 (1.40)	0.13	–0.02	0.06	0.16							
(6) Positive emotion	3.47 (1.34)	–0.19^†^	0.14	0.14	0.20^†^	–0.15						
(7) Focus on self	4.74 (1.25)	0.22*	–0.12	–0.20^†^	0.45***	0.27**	0.22*					
(8) Focus on judge	4.91 (1.23)	0.06	–0.14	–0.07	0.41***	0.31**	–0.09	0.32**				
(9) Self-evaluation	5.87 (1.47)	–0.05	0.10	0.21^†^	0.24*	–0.33**	0.42***	0.11	0.15			
(10) Metaperception	5.53 (1.51)	–0.05	0.18^†^	0.27*	0.29**	–0.37***	0.45***	0.07	0.04	0.82***		
(11) Judge’s evaluation	6.72 (0.94)	0.13	0.19^†^	0.04	0.07	–0.19^†^	0.14	0.20^†^	–0.08	0.25*	0.37***	
(12) Optimistically biased metaperception	–1.18 (1.46)	–0.14	0.06	0.26*	0.25*	–0.27*	0.37***	–0.06	0.09	0.69***	0.80***	–0.27*

*^∗^*p* < 0.05, ^∗∗^*p* < 0.01, ^∗∗∗^*p* < 0.001, ^†^*p* < 0.10. Optimistically biased metaperception = metaperception – judge’s evaluation.*

We also tested if gender differences existed in social interaction with this male judge. As a result, gender was not associated with eagerness, negative emotion, focus on judge, self-evaluation, metaperception, judge’s evaluation, or optimistically biased metaperception, *p*s > 0.180. It was correlated with focus on self (*r* = 0.22, *p* = 0.043) and marginally correlated with positive emotion (*r* = -0.19, *p* = 0.082). These results suggested that participants’ gender, in general, did not influence their performances.

More importantly, eagerness was correlated with self-evaluation (*r* = 0.24, *p* = 0.028), metaperception (*r* = 0.29, *p* = 0.006), and optimistically biased metaperception (*r* = 0.25, *p* = 0.018), but not with the evaluation of the judge (*r* = 0.07, *p* = 0.499). These results showed that self-evaluation, metaperception, and optimistically biased metaperception increased with eagerness.

Eagerness was also correlated with positive emotion (*r* = 0.20, *p* = 0.068), focus on self (*r* = 0.45, *p* < 0.001), and focus on the judge (*r* = 0.41, *p* < 0.001). These findings indicated that highly eager participants tended to have a broad focus and experience intense positive emotions. However, eagerness was not related to negative emotion (*r* = 0.16, *p* = 0.133).

Consistent with feelings-as-information theory, positive emotion was positively correlated with self-evaluation (*r* = 0.42, *p* < 0.001), metaperception (*r* = 0.45, *p* < 0.001), and optimistically biased metaperception (*r* = 0.37, *p* < 0.001), whereas negative emotion was negatively correlated with self-evaluation (*r* = -0.33, *p* = 0.002), metaperception (*r* = -0.37, *p* < 0.001), and optimistically biased metaperception (*r* = -0.027, *p* = 0.012). However, neither focus on self nor focus on the judge was correlated with self-evaluation, metaperception, or optimistically biased metaperception, *p*s > 0.160.

**FIGURE 1:**
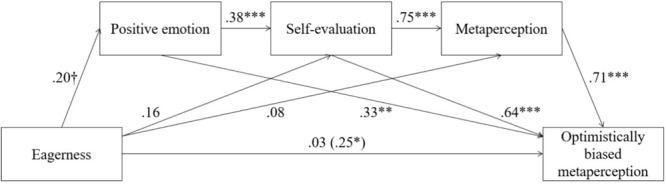
Multistep mediation model (Study 2). Standardized coefficients and their corresponding significance are reported. Total effects are denoted in parentheses. The standardized coefficients when the mediator is included in the model are presented above the arrow. ^∗^*p* < 0.05, ^∗∗^*p* < 0.01, ^∗∗∗^*p* < 0.001, ^†^*p* < 0.10. Optimistically biased metaperception = metaperception – judge’s evaluation.

A mediation analysis (Model 6, Hayes and Preacher, 2014) was then conducted, with eagerness as the independent variable, positive emotion, self-evaluation, and metaperception as the mediators, and optimistically biased metaperception as the dependent variable (**Figure 1**). The analysis based on 5,000 bootstrap samples generated a 95% CI excluding zero for this pathway ([0.01, 0.12]). This finding indicated that eagerness influenced optimistically biased metaperception through intense positive emotions, increased self-evaluations, and increased metaperceptions sequentially.

However, these effects are correlational in nature and are thus prone to questions of causality. Because the participants indicated their eagerness after their performance on the task, it was possible that participants’ self-evaluation was high and they experienced positive emotion because they felt they had done well. Thus, we tested some theoretically sound competing mediation models (**Table 3**). As a result, all these models were invalid statistically. Therefore, positive emotion in this study was not caused by self-evaluation (Model 2 in **Table 3**). In addition, eagerness cannot be attributed to positive emotion induced by self-evaluation (Models 3 and 4 in **Table 3**).

**Table 3:** Multiple pathways (Study 2).

	Model	Confidence interval
1	Eagerness → positive emotion → self-evaluation → metaperception → optimistically biased metaperception	[0.01, 0.12]
2	Eagerness → self-evaluation → metaperception → optimistically biased metaperception → positive emotion	[–0.04, 0.05]
3	Self-evaluation → metaperception → optimistically biased metaperception → positive emotion → eagerness	[–0.02, 0.03]
4	Self-evaluation → positive emotion → eagerness → metaperception → optimistically biased metaperception	[–0.01, 0.02]

*Optimistically biased metaperception = metaperception – judge’s evaluation.*

Similar to Study 1, the result that eagerness was not significantly correlated with the judge’ evaluation (*r* = 0.07, *p* = 0.499) excluded the alternative explanation that participants who believed that their performances were objectively better were more eager to learn their results.

In summary, the relationship between eagerness and optimistically biased metaperceptions and the roles of emotional experiences and cognitive focus were explored in a well-controlled setting. The results showed that eagerness was correlated with optimistically biased metaperceptions because it induced positive emotions, increased self-evaluations, and increased metaperceptions. In this study, we provided evidence for our hypothesized pathway (i.e., eagerness → positive emotion → self-evaluation → metaperception → optimistically biased metaperception) and ruled out some competing pathways statistically. In Study 3, we tried to manipulate eagerness.

## Study 3: Interview

In this study, we attempted to investigate the casual relationship between eagerness and optimistically biased metaperceptions. To our knowledge, however, there was no existing well-acknowledged paradigm in manipulating eagerness. Our logic was as follows. The participants were interviewed by a well-trained interviewer. Half participants were told that the interview was effective in assessing interviewees’ communication skill. Another half was told that the interview cannot precisely show interviewees’ communication skill. The difference in validity would evoke different levels of eagerness to find out interviewers’ evaluations.

### Method

#### Participants and Design

We recruited 102 university students who were randomly assigned to a high or low eagerness condition. Ten participants were excluded because they did not complete the interview. The final sample comprised 92 participants (22 men, 70 women; *M*_age_ = 20.33 years, *SD* = 2.19; *N*_low_ = 43, *N*_high_ = 49). We assumed a medium effect size (Cohen’s *d* = 0.50) of the relationship between eagerness and optimistically biased metaperception, the power of this study is about 67%, given the sample size of 92 and α = 0.05 (Cohen, 1970).

#### Procedure and Materials

The participants arrived at Laboratory A, where an experimenter instructed them to undergo an interview to assess their interpersonal communication skill. In the high eagerness condition, the participants were informed that the interview was developed by psychology professors and human resource managers. This interview has been adopted in several famous corporations because of its effectiveness in assessing the communication skill of interviewees. In the low eagerness condition, participants were informed that the interview was being developed by undergraduate and graduate students in psychology. This interview did not precisely show their communication skill and required further tests to increase its effectiveness.

The participants were then led to Laboratory B, where they were interviewed by the same female interviewer. The interviewer was not aware of the research aim and the grouping of the participants. The interview was semi-structured. The participants were asked to describe themselves, a partner with whom they once worked together with, and their experiences with the partner. To avoid unnecessary feedback, the interviewer spoke in a neutral tone during the interviews. She maintained neutral facial expressions and avoided body language. Her behavior was standardized for all the participants. The interviews were videotaped, which indicated the length of interview for each participant.

The participants returned to Laboratory A after the interviews and then completed a questionnaire. First, they rated their eagerness (“I am eager to know the interviewer’s evaluation of me,” “I care about the interviewer’s evaluation of me,” and “The interviewer’s evaluation of me is important to me;” α = 0.81; 1 = *totally disagree*, 7 = *totally agree*). They then judged how the interviewer evaluated their interpersonal communication skill (i.e., metaperception; 0 = *very poor*, 10 = *very good*).

Because previous research has yielded an effect of perspective taking on metaperception (Vorauer et al., 2009), the level of perspective taking was assessed by the perspective-taking subscale of interpersonal reactivity index, which is composed of seven items (e.g., “Before criticizing somebody, I try to imagine how I would feel if I were in their place”; α = 0.79; 1 = *does not describe me well*, 5 = *describes me very well*) (Davis, 1980). Next, the participants indicated their gender and age and were then finally debriefed and paid for their participation.

The interviewer stayed in Laboratory B and rated the communication skill of each participant (0 = *very poor*, 10 = *very good*). The experimenter paired the interviewer’s evaluations and the participants’ ratings.

### Results and Discussion

Participants in the high eagerness condition (*M* = 4.95, *SD* = 1.25) reported higher eagerness than the participants in the low eagerness condition (*M* = 4.34, *SD* = 1.31), *t*(90) = -2.29, *p* = 0.025, Cohen’s *d* = -0.48, demonstrating a successful manipulation.

The length of the interview (*M*_high_ = 277.27 s, *SD* = 85.03; *M*_low_ = 284.74 s, *SD* = 92.76) and the level of perspective taking (*M*_high_ = 3.62, *SD* = 0.57; *M*_low_ = 3.47, *SD* = 0.65) did not differ for participants in the high and low eagerness conditions, *t*(90) = 0.40, *p* = 0.688, Cohen’s *d* = 0.08 and *t*(89) = -1.21, *p* = 0.229, Cohen’s *d* = -0.25^2^, respectively. Hence, these two variables were excluded in the subsequent statistical analysis.

The rating of the interviewer was subtracted from the metaperception of each participant to reflect an optimistically biased metaperception. The optimistically biased metaperception was marginally higher in the high eagerness condition (*M* = 1.15, *SD* = 2.03) than in the low eagerness condition (*M* = 0.35, *SD* = 2.18), *t*(90) = -1.83, *p* = 0.070, Cohen’s *d* = -0.38. This result suggested that eagerness led to optimistically biased metaperceptions.

Finally, we investigated gender difference. The results showed that eagerness, metaperception, interviewer’s evaluation, and optimistically biased metaperception were unaffected by participants’ gender, *p*s > 0.050.

## General Discussion

People commonly fail to produce accurate social predictions (Van Boven et al., 1999; Epley and Dunning, 2001). In this study, we focus on metaperception, a specific type of social prediction. Individuals exert considerable effort to determine accurate metaperceptions throughout their lifetime because they are beneficial to good social relationships (Carlson, 2016) and psychological well-being (Human et al., 2013). The current study investigates the relationship between eagerness to learn other’s evaluations and optimistically biased metaperceptions. We show that a high level of eagerness biases metaperceptions. When a person is highly eager to know how others rate him or her, he or she will experience intense positive emotions. These positive emotions cause optimistic self-perceptions, which result in high metaperceptions.

### Theoretical and Practical Implications

The current results contribute to the existing literature on social prediction. Researchers rely largely on introspection illusion, spotlight, or empathy gap theory to understand why people fail to make accurate social inferences. All these theories propose an egocentric account for biased social predictions. However, none can provide an explanation for the effect of eagerness on biased metaperceptions.

We adopt a “motivation–emotion–cognition” perspective to deconstruct why eagerness influences metaperceptions, a form of cognition. In accordance with this view, motivational, emotional, and cognitive factors interact with one another. Eagerness, a motivational factor, induces positive emotions. Although these emotions are incidental, they play an important role in forming metaperceptions, a finding that is in line with feelings-as-information theory (Schwarz, 2012).

However, our results do not suggest that metaperceptions are not egocentrically biased when people are highly eager to know others’ evaluations, because Studies 1 and 2 show a high correlation between self-evaluations and metaperceptions, revealing egocentric biased metaperceptions regardless of the level of eagerness. What the current study reveals is that eagerness influences emotional experiences, which in turn increase self-evaluations and metaperceptions.

In Study 2, we pit against cognitive and emotional routes to determine why eagerness influences metaperceptions. Our results show that the emotional path is dominant to cognitive path in the relationship between eagerness and metaperceptions. We reveal that cognitive processes are not necessary in shaping metaperceptions. Eagerness can affect metaperception even without changing the cognitive focus.

The current research sheds light on the effect of emotion on judgment and decision making. Judgment and choice are influenced by the incidental emotions people experience when making a decision (Peters et al., 2006). Individuals attend to incidental emotions as sources of information in judging. For instance, anger and fear exert opposite effects on risk perception (Lerner and Keltner, 2000). In our study, self-evaluations and metaperceptions are determined by the intensity of positive emotions. Pfister and Böhm (2008) claimed a fourfold function of emotions on judgment and decision making. Emotions provide information, enable rapid decisions, direct decision makers’ attention, and generate commitment. Our research pays further attention to how emotions provide information and direct attention in forming self-evaluations and metaperceptions. Researchers could focus on the other two functions in the future.

Our results also carry practical implications. The “more–more” pattern (i.e., more efforts, better outputs) regarding the link between effort and output is sometimes an illusion. At least, this pattern does not apply to the link between eagerness and accurate metaperceptions. In accordance with our results, when developing a romantic relationship, negotiating with partners, or taking part in an interview, high eagerness should be avoided to prevent overoptimistic metaperceptions.

### Limitations and Future Directions

One of the strengths of this research is that it is a mixture of field and laboratory studies. We did not control for the behavior of the judges in Study 1 to increase external validity and show the relationship between eagerness and optimistically biased metaperceptions in a natural setting. Only one judge was employed in Studies 2 and 3 to solve this problem, because showing identical facial expressions to all participants is easier for one judge than for multiple judges. Nevertheless, their facial expressions still might not have been completely identical across participants. These inconsistencies might affect the influence of eagerness on optimistically biased metaperceptions. In future studies, researchers should inform participants that they will interact with a judge through a real-time video, but, in reality, the participants will watch a pre-taped video. However, these measures may weaken external validity.

One may argue that people who are highly eager to know how others rate them are motivated to receive positive feedback. Therefore, high metaperceptions result from a self-serving bias or a self-protection mechanism (Preuss and Alicke, 2009; Carlson, 2013). The results in Study 1 demonstrated a correlation between eagerness to know the evaluations of others and motivations to obtain a high score (**Table 1**). However, the former predicted optimistically biased metaperceptions, whereas the latter did not, thereby excluding this explanation.

Another concern involves the generalizability of our results. Singers in a singing contest on average had a high eagerness to know how they were rated by judges (*M* = 5.12 on a seven-point scale). However, people in daily social interactions are not necessarily that eager to know others’ evaluations of themselves. Study 2 remedied this limitation because participants were required to deliver a speech (rather than initiatively delivered a speech). Their eagerness was, therefore, moderate (*M* = 4.31 on a seven-point scale). A related question is whether awareness of eagerness will influence the observed relationship between eagerness and metaperceptions. We speculate that it won’t. Eagerness should cause positive emotions, optimistic evaluations and high metaperceptions, regardless of awareness.

Moreover, as in most previous studies, participants were engaged in artificial interpersonal interactions in all three tasks in our studies where participants were clearly aware that they were rated by others. Although it captures some types of social interactions in real life (e.g., taking part in a job interview), this awareness is not salient in others (e.g., chatting with a friend). Carlson et al. (2011) found more biased metaperceptions in artificial than naturalistic interactions. Therefore, future research test if our findings can be generalized to real-life situations.

Finally, Study 3 served as an exploratory attempt to manipulate eagerness to learn others’ evaluations. Additional evidence is needed to show its reliability and validity. Furthermore, we should treat this result with caution because of its marginal significance.

## Conclusion

This research corroborates that optimistically biased metaperceptions are influenced by the eagerness to know the evaluations of others. High eagerness leads to optimistically biased metaperceptions through increased positive emotions, optimistic self-evaluations, and increased metaperceptions.

## Author Contributions

JL and XX developed the concepts for the study. JL and HD collected the data and analyzed the data. All authors contributed to the manuscript and approved the final version of the manuscript for submission.

## Conflict of Interest Statement

The authors declare that the research was conducted in the absence of any commercial or financial relationships that could be construed as a potential conflict of interest.

## Footnotes

1 Some of these participants dropped out, whereas others delivered a speech that was less than 120 s. Thus, the judge was unable to nod or shake his head as required (see “Procedures and materials” for details).

2 There was one missing value.
